# Suppression of Gut Bacterial Translocation Ameliorates Vascular Calcification through Inhibiting Toll-Like Receptor 9-Mediated BMP-2 Expression

**DOI:** 10.1155/2019/3415682

**Published:** 2019-03-17

**Authors:** Yang Zhao, Yan Cai, Li-Yan Cui, Wen Tang, Bo Liu, Jia-Jia Zheng, Wen-Zhe Si, Xian Wang, Ming-Jiang Xu

**Affiliations:** ^1^Department of Laboratory Medicine, Peking University Third Hospital, Beijing 100191, China; ^2^Department of Physiology and Pathophysiology, School of Basic Medical Science, Peking University Health Science Center, Key Laboratory of Molecular Cardiovascular Science, Ministry of Education, Beijing 100191, China; ^3^School of Pharmaceutical Sciences, Wenzhou Medical University, Wenzhou, Zhejiang, China; ^4^Department of Nephrology, Peking University Third Hospital, Beijing 100191, China

## Abstract

**Aims:**

Vascular calcification (VC) is a primary risk factor for cardiovascular mortality in chronic renal failure (CRF) patients; thus, effective therapeutic targets are urgently needed to be explored. Here, we identified the role of intestinal bacterial translocation in CRF-related VC.

**Methods and Results:**

Antibiotic supplementation by oral gavage significantly suppressed intestinal bacterial translocation, CRF-related VC, and aortic osteogenic gene and Toll-like receptor (TLR) gene expression in CRF rats. Furthermore, TLR4 and TLR9 activation in vascular smooth muscle cells (VSMCs) aggravated inorganic phosphate- (Pi-) induced calcification. TLR9 inhibition, but not TLR4 inhibition, by both a pharmacological inhibitor and genetic methods could significantly reduce CRF rats' serum or CRF-induced VC. Interestingly, bone morphogenic protein-2 (BMP-2) levels were increased in the aorta and sera from CRF rats. Increased BMP-2 levels were also observed in VSMCs treated with TLR9 agonist, which was blocked by NF-*κ*B inhibition. Both siRNA knockdown of BMP-2 and NF-*κ*B inhibitor significantly blocked TLR9 agonist-induced VSMC calcification.

**Conclusions:**

Gut bacterial translocation inhibited by oral antibiotic significantly reduces CRF-related VC through inhibition of TLR9/NF-*κ*B/BMP-2 signaling.

## 1. Introduction

Vascular calcification (VC) is the major cardiovascular complication in chronic renal failure (CRF) patients. The risk factors for VC in CRF patients include renal function decline, disordered mineral metabolism, and systemic inflammation [1]. Systemic inflammation is a common feature of CRF patients and closely related to morbidity and cardiovascular events [2]. Recently, accumulating evidence has demonstrated that the gastrointestinal tract is a major instigator of systemic inflammation in CRF [3]. Studies illustrated that increased intestinal permeability due to intestinal barrier dysfunction induces intestinal bacterial translocation in both CRF patients and experimental CRF models [4–6]. The colon wall inflammation is along with destruction of the intestinal epithelial tight junction barrier, which leads to translocation of bacterial DNA and lipopolysaccharide (LPS) into bloodstream. Gut bacterial DNA and LPS can be detected in the serum of CRF animals and dialysis patients and correlate with severity of systemic inflammation, suggesting that intestinal bacterial translocation is an important cause of systemic inflammatory response in CRF [3, 4].

Bacterial translocation has been discovered in multiple diseases such as chronic liver disease, kidney injury, and atherosclerosis, during which LPS- and bacterial DNA-induced immune responses are the leading cause of organ damage [7–11]. TLR4 and TLR9, as the receptors for bacterial LPS and bacterial DNA, respectively, are involved in the morbidity and development of these diseases. Activation of the LPS/TLR4 signal increases the generation of reactive oxygen species and inflammatory cytokines in arterial endothelial cells and VSMCs [12]. Monocyte/macrophages (M/Ms), activated by LPS, promote osteoblastic differentiation and mineralization of calcifying vascular cells (CVCs) [13], while bacterial DNA/TLR9 signaling promotes atherosclerosis [14] and vascular endothelial injury [9]. These data indicates a relationship between TLR4/TLR9 signaling and inflammatory vascular disease.

However, whether bacterial components contribute to the inflammation and VC in CRF individuals remains unknown. In the present study, oral antibiotic were used to suppress intestinal bacteria and its product LPS and bacterial DNA in CRF rats. We demonstrate that antibiotic administration alleviates intestinal bacterial translocation and suppresses vascular calcification in adenine-induced CRF rats through inhibition of TLR9/NF-*κ*B/BMP-2 signaling.

## 2. Methods

### 2.1. Animal Protocols for the Rat Model

All animals received humane care in compliance with the Institutional Authority for Laboratory Animal Care of Peking University which complies with the Guide for the Care and Use of Laboratory Animals published by the US National Institutes of Health (NIH Publication No. 85-23, revised 1996). The adenine-induced CRF rat model was established as described previously [15–17]. 8-week-old male Wistar rats were pair-fed with standard chow containing 1.2% calcium and 0.6% phosphorus for the control group or 0.75% adenine and 1.0% phosphorus for the CRF and CRF + Anti groups for 6 weeks. Polymycin B sulfate (150 mg/kg·day per rat; Aladdin, Shanghai, China) and neomycin sulfate (450 mg/kg·day per rat; Aladdin, Shanghai, China) were given intragastrically for 6 weeks. For euthanasia, sodium pentobarbital (45 mg/kg, intraperitoneal injection) was used to anaesthetize the rats, the blood was drawn from the rat inferior vena cava, and then the rats were decapitated; the aortas were excised, and efforts were made to minimize animal suffering.

Abdominal aortas were excised and fixed in 4% formaldehyde and sectioned for von Kossa staining. For measurement of calcium content, the abdominal aortas were dried and weighed, each sample was extracted with 65% (*w*/*w*) HNO_3_ for 24 h at 200°C to dissolve the minerals, and the calcium content was measured by atomic absorption spectrometry at 422.7 nm (Jena, novAA 300, Jena, Germany). Results were normalized by dry tissue weight.

### 2.2. Animal Protocols for the Mouse Model

TLR4^−/−^ and TLR9^−/−^ mice were purchased from Nanjing Biomedical Research Institute of Nanjing University (NBRI), and the genetic background is C57BL/6J. The adenine diet-induced CRF mouse model was applied as we described previously [18]. 20% casein was added to the chow diet and adenine diet to cover the taste and smell of adenine. The chow diet contains 0.6% phosphate, and the adenine diet contains 1.0% phosphate. 8-week-old male c57, TLR4^−/−^, or TLR9^−/−^ mice were randomly divided into chow diet (Ctrl) and adenine diet (CRF) groups. Mice were housed under constant temperature (23 + 1°C) with a 12 h light and 12 h dark cycle with free access to water and chow and sacrificed by cervical dislocation. After an 8-week diet program, the calcium content of abdominal aortas was measured.

### 2.3. Aortic Ring Calcification

Thoracic aortas were removed in a sterile manner from 8-week-old c57, TLR4^−/−^, and TLR9^−/−^ mice. After the adventitia and endothelium were carefully removed, the vessels were cut into ~1 mm rings and placed in a high-Pi (3 mmol/L) or normal culture medium at 37°C in 5% CO_2_ for 7 days. The medium was replaced every 3 days.

### 2.4. Cell Culture and Cell Calcification Model

T/G human aortic smooth muscle cells (HASMCs) transfected with scramble small interferon RNA (C-siR) or BMP-2-siRNA (sense, 5′-GCAACAGCCAACUCGAAAUdTdT-3′; antisense, 5′-AUUUCGAGUUGGCUGUUGCdTdT-3′) were from GenePharma (Shanghai, China) designed by use of the Block-iT™ RNAi Designer. HASMCs were cultured as we described [16]. For calcification experiments, cells were seeded at 1 × 10^4^ cells/cm^2^ (day 0) and maintained in 10% fetal bovine serum/DMEM until confluence (day 6), when calcification was induced by adding 3.0 mmol/L Pi. After 7 days of inducing calcification, calcium deposit was detected by measuring the calcium content as we described [15–17, 19]. For treatment of cells, ssDNA, TLR9 ligand control, TLR9 inhibitor, TLR4 inhibitor (Invivogen, San Diego, CA), parthenolide (Calbiochem, Germany), and LPS (Sigma, St Louis, MO) were added with Pi every 3 days.

### 2.5. Western Blot Analysis

Following treatment, the relative cells and aortic extracts were collected. Proteins were subjected to sodium dodecyl sulfate polyacrylamide gel electrophoresis and then transferred onto a nitrocellulose membrane, then incubated successively with 3% bovine serum albumin and different primary antibodies: anti-Cbf*α*-1 (1 : 1000, CST, Danvers, MA), anti-I*κ*B*α*, anti-BMP-2, anti-SM22*α*, anti-GAPDH, and anti-*β*-actin (1 : 1000, all from Santa Cruz Biotechnology, Santa Cruz, CA). The membranes were incubated in IRDye® 700 or 800-conjugated secondary antibody (1 : 20000, Rockland Immunochemicals Inc., Gilbertsville, PA) for 1 hr. The fluorescence signal was then detected using the Odyssey infrared imaging system (LI-COR Biosciences, Lincoln, NE).

### 2.6. Real-Time PCR Analysis

Total RNA from aortic tissue or cells was isolated using TRIzol and subjected to a reverse transcription system (Promega, Madison, WI). For real-time PCR, 1 *μ*L of the reaction mixture was used. The amount of PCR products formed in each cycle was evaluated by SYBR Green I fluorescence (Invitrogen, Carlsbad, CA). The forward and reverse PCR primers based on rat genes were *Cbfα-1*, 5′-ACTACTCTGCCGAGCTACGA-3′, 5′-GCCACTTGGGGAGGATTTGT-3′; *Msx-*2, 5′-GGAGATTGCAAGAGGGCGTA-3′, 5′-GGGCTAGCTGACTGTGTTGT-3′; *SM22α*, 5′-GTTTGGCCGTGACCAAGAAC-3′, 5′-AAGCTGTCCGGGCTAAGAAC-3′; *α-actin*, 5′-AGGAGTATGACGAAGCTGGC-3′, 5′-GAAAAGAACTGAAGGCGCTGA-3′; *TLR4*, 5′-CTACCTCGAGTGGGAGGACA-3′, 5′-TGCTACTTCCTTGTGCCCTG-3′; *TLR9*, 5′-GCCCCAGAACCTCAACTACC-3′, 5′-AAACCAGGAGCGATCCACAG-3′; and *β-actin*, 5′-GAGACCTTCAACACCCCAGCC-3′, 5′-TCGGGGCATCGGAACCGCTCA-3′. The forward and reverse PCR primers based on human genes were *Cbfα-1*, 5′-CGCCTCACAAACAACCACAG-3′, 5′-TCACTGTGCTGAAGAGGCTG-3′; *SM22α*, 5′-GGAGCTTGCGGGAAGGATTA-3′, 5′-CCATTGCCTTCCTGTTGCAC-3′; *β-actin*, 5′-ATCTGGCACCACACCTTC-3′, 5′-AGCCAGGTCCAGACGCA-3′; *BMP-2*, 5′-CGTCAACTCGATGCTGTACCT-3′, 5′-CAACCCTCCACAACCATGTCC-3′; and *α-actin*, 5′-GCCAAGCACTGTACAGGAATC-3′, 5′-CACCATCACCCCCTGATGTC-3′. Serum bacterial DNA was extracted using the QIAamp DNA Mini Kit (Qiagen, GmbH, Germany) according to the manufacturer's protocol. The forward primer was SDBact-0008-a-S-20 (5′-AGAGTTTGATCCTGGCTCAG-3′), which targets the domain bacteria, and the reverse primer was SUniv0536 (5′-GWATTACCGCGGCKGCTG-3′). All amplification reactions involved use of the Mx3000 Multiplex Quantitative PCR System (Stratagene, La Jolla, CA).

### 2.7. Statistical Analysis

All data are presented as mean ± SEM unless otherwise stated. Data was analyzed by the use of GraphPad Prism software. Statistical analysis involved one-way ANOVA for multiple comparisons, then Tukey-Kramer post hoc testing, and Student's unpaired *t*-test for comparisons between two groups. *P* < 0.05 was considered as statistically significant.

## 3. Results

### 3.1. Antibiotic Administration Inhibits Intestinal Bacterial Translocation in CRF Rats

**Figure 1:**
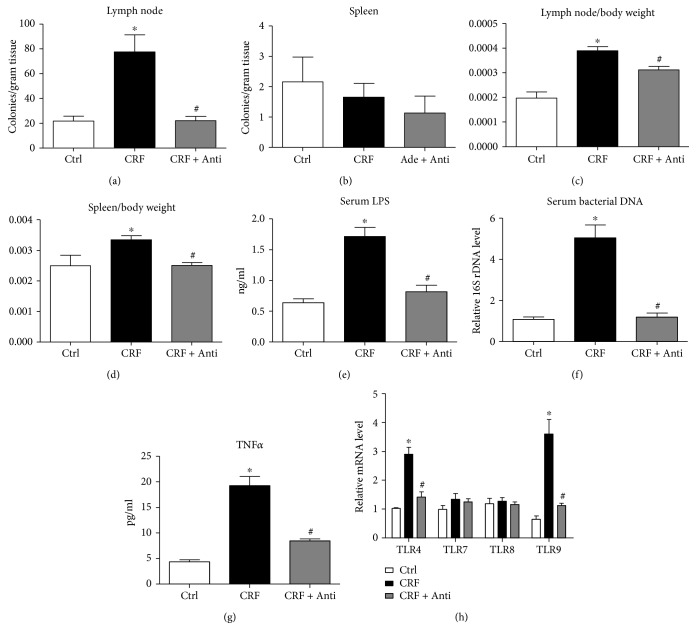
Bacteria translocation in adenine-induced CRF rats. (a-b) The mesenteric lymph node and spleen from Ctrl, CRF, and CRF plus antibiotics (CRF + Anti) rats were grinded with PBS and cultured on blood agar at 37°C for 36 h, and the colony number per gram tissue was calculated. (c-d) The mesenteric lymph node weight/body weight and spleen weight/body weight of Ctrl, CRF, and CRF + Anti rats were calculated. (e) Chromogenic End-point TAL Kit detected serum LPS levels in Ctrl, CRF, and CRF + Anti rats. (f) Serum bacterial DNA of Ctrl, CRF, and CRF + Anti rats was extracted using the QIAamp DNA Mini Kit, and real-time PCR analyzed the 16S rDNA level. (g) Serum TNF*α* levels of Ctrl, CRF, and CRF + Anti rats were measured by ELISA. (h) Thoracic aorta mRNA was extracted from Ctrl, CRF, and CRF + Anti rats, and real-time PCR analyzed the mRNA levels of TLR4, TLR7, TLR8, and TLR9. *n* = 8~12, ^∗^ *P* < 0.05 vs. Ctrl, ^#^ *P* < 0.05 vs. CRF.

To investigate intestinal bacterial translocation and its harmful product on CRF-induced VC, polymyxin B sulfate and neomycin sulfate were administered orally. The lymph node tissue and spleen tissue homogenate were cultured on a blood agar plate for 36 h. The colony number of bacteria per gram tissue was significantly increased in the mesenteric lymph node of CRF rats and reduced to normal level in antibiotic treatment rats; there was no significant difference in bacteria number in the spleen tissue among the three groups (Figures 1(a) and 1(b)). The mesenteric lymph node/body weight and spleen/body weight ratios were increased in CRF rats compared with control rats, which were reversed by antibiotic treatment (Figures 1(c) and 1(d)). Similarly, antibiotic administration significantly reduced serum LPS and bacterial DNA levels in CRF rats (Figures 1(e) and 1(f)). As expected, serum TNF*α* increased in CRF rats which was reversed to control level after treatment with antibiotics (Figure 1(g)). In addition, we found that TLR4 and TLR9, two receptors for LPS and bacterial DNA, respectively, were elevated in the aortic tissue from CRF rats, and they were decreased to normal levels with antibiotic treatment (Figure 1(h)). However, antibiotic administration did not improve adenine-induced renal failure and hyperphosphatemia except a little decrease in serum creatinine (Supplementary Figure 1).

### 3.2. Antibiotics Reduce Vascular Calcification in CRF Rats

**Figure 2:**
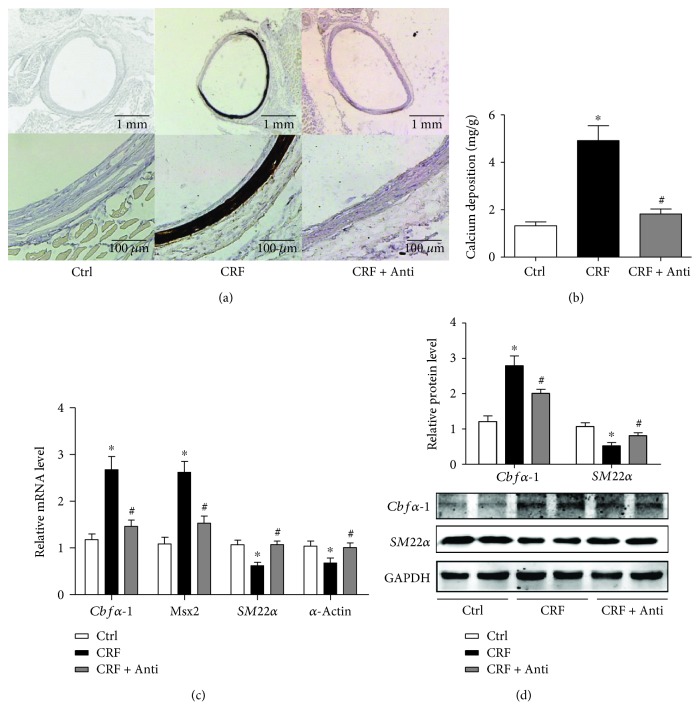
Antibiotics reduce vascular calcification and inflammation in adenine-induced CRF rats. (a) von Kossa staining of the rat abdominal aorta of Ctrl, CRF, and CRF + Anti rats. (b) Calcium content of abdominal aorta was measured and normalized by dried tissue weight. (c) Real-time PCR analyzed the mRNA levels of osteogenic gene s*core-binding factor α-1* (*Cbfα1*), *msh homeobox 2* (*Msx2*); VSMC lineage markers *SM22α* and *α*-actin in the rat aorta from Ctrl, CRF, and CRF + Anti groups. Data are relative to the *β*-actin level. (d) Western blot analyses of *Cbfα1* and *SM22α* protein levels in the rat aorta from Ctrl, CRF, and CRF + Anti groups. GAPDH was a loading control.*n* = 8~12, ^∗^ *P* < 0.05 vs. Ctrl, ^#^ *P* < 0.05 vs. CRF.

Calcium deposition in the abdominal aorta, as assessed by von Kossa staining and calcium content assay, was increased in CRF rats, and antibiotic administration significantly ameliorated the calcium deposition (Figures 2(a) and 2(b)). Antibiotic administration significantly reduced the mRNA levels of osteogenic genes Msx2 and Cbf*α*-1 and increased smooth muscle lineage markers Actin2 and SM22*α* compared with those in vehicle-administered CRF rats' aorta (Figure 2(c)). These observations were further confirmed by western blot that Cbf*α*-1 protein increased while SM22*α* diminished in CRF rat aorta and antibiotic administration reversed these changes (Figure 2(d)).

### 3.3. Bacterial Components LPS and DNA Promote Pi-Induced Calcification and Osteoblastic Differentiation in HASMCs

**Figure 3:**
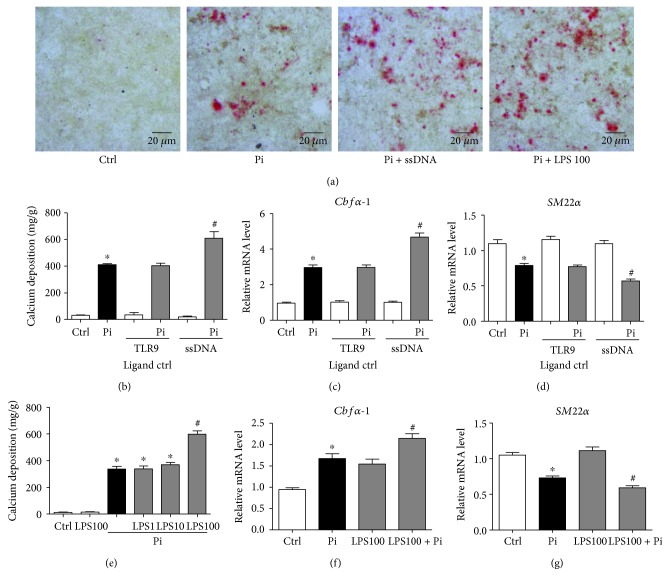
Bacterial components LPS and DNA promote Pi-induced calcification and osteoblastic differentiation in HASMCs. HASMCs were treated with inorganic phosphate (Pi), E. coli ssDNA (10 *μ*g/mL), or LPS (100 ng/mL) as indicated for 7 days, and alizarin red staining was operated. (b-d) HASMCs were treated with TLR9 ligand control (1 *μ*g/mL) or E. coli ssDNA (ssDNA, 10 *μ*g/mL), with or without Pi for 7 days. (b) Calcium content was measured. Real-time PCR analyzed the mRNA levels of (c) *Cbfα1* and (d) *SM22α*. Data are relative to *β-actin* level. (e-g) HASMCs were treated with LPS at the indicated concentrations (ng/mL), with or without Pi for 7 days. (e) Calcium content was measured. Real-time PCR analyzed the mRNA levels of (f) *Cbfα1* and (g) *SM22α.* Data are relative to *β-actin* level. *n* = 3, ^∗^ *P* < 0.05 vs. Ctrl, ^#^ *P* < 0.05 vs. Pi.

As TLR4 and TLR9 are expressed in HASMCs responsive to their ligands [11], we aimed to test the effect of TLR4 and TLR9 signaling activation on Pi-induced VSMC calcification. Human aortic smooth muscle cells (HASMCs) were treated with TLR9 ligand control (a ligand binding to TLR9 but cannot activate it) or TLR9 activator E. coli ssDNA (ssDNA) or TLR4 activator LPS at the indicated concentration (ng/mL), with or without Pi (3.0 mmol/L) for 24 h or 7 days. Alizarin red staining revealed that ssDNA and LPS augmented Pi-induced HASMC calcification (Figure 3(a)). TLR9 activation significantly increased calcium content in Pi-treated HASMCs (Figure 3(b)), which was accompanied with remarkably upregulated Cbf*α*-1 and downregulated SM22*α* gene expression compared with the ligand control group in Pi-treated HASMCs (Figures 3(c) and 3(d)). Similar results were observed in high-dose LPS-treated HASMCs (Figures 3(e)–3(g)).

### 3.4. Antibiotics Ameliorate Vascular Calcification in CRF Rats through Reducing TLR9 Signaling

**Figure 4:**
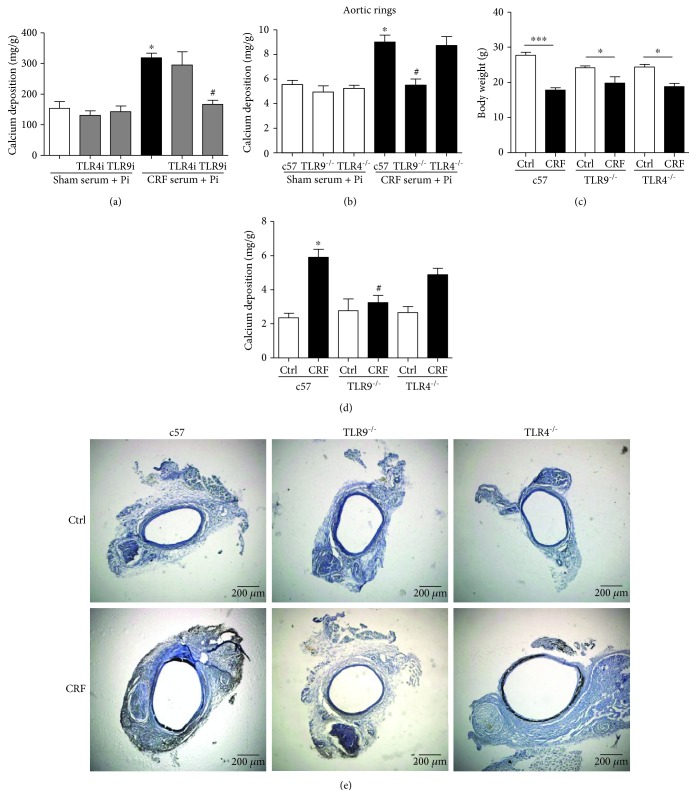
Antibiotics ameliorate vascular calcification in CRF rats through TLR9 signaling. (a) HASMCs were treated with sham rats' serum or CRF rats' serum, with or without TLR4 inhibitor (1 *μ*g/mL, preincubated for 30 min) or TLR9 inhibitor (1 *μ*mol/L, preincubated for 30 min), and then treated with Pi for 7 days. Calcium content was measured (*n* = 3, ^∗^ *P* < 0.05 vs. sham serum + Pi, ^#^ *P* < 0.05 vs. CRF serum + Pi). (b-d) Male 8-week-old c57 mice, TLR4^−/−^, mice and TLR9^−/−^ mice were fed with high-casein diet (Ctrl) or high-casein diet plus adenine (CRF) for 8 weeks. (b) Aortic rings of c57, TLR4^−/−^ mice, and TLR9^−/−^ mice were cultured and treated with sham rats' serum or CRF rats' serum and then treated with Pi for 7 days. Calcium content was measured. *n* = 3, ^∗^ *P* < 0.05 vs. c57 + sham serum + Pi, #*P* < 0.05 vs. c57 + CRF serum + Pi. (c) Body weight was measured (*n* = 6~11, ^∗^ *P* < 0.05, ^∗∗∗^ *P* < 0.0001). (d) Calcium content of abdominal aorta was measured and normalized by dried tissue weight. *n* = 6~11, ^∗^ *P* < 0.05 vs. c57 Ctrl, #*P* < 0.05 vs. c57 CRF. (e) von Kossa staining of abdominal aorta from c57, TLR4^−/−^, and TLR9^−/−^ mice fed with high-casein diet (Ctrl) or high-casein diet plus adenine (CRF).

Pi-treated HASMCs were preincubated with PBS, TLR4 inhibitor, or TLR9 inhibitor for 30 min and then treated with serum from control rats or CRF rats. CRF rat serum augmented calcium deposition in Pi-treated HASMCs compared with control rat serum, which was notably alleviated by TLR9 inhibition but not TLR4 inhibition (Figure 4(a)). However, both TLR4 and TLR9 inhibitors could significantly reduce CRF rat serum-induced inflammatory cytokine secretion in primary macrophages (Supplementary Figure 2). Furthermore, aortic rings from WT, TLR4^−/−^, and TLR9^−/−^ mice were treated with Pi plus serum from control rats or CRF rats. CRF rat serum significantly increased Pi-induced calcium deposition in WT and TLR4^−/−^ aorta, but not in TLR9^−/−^ aorta (Figure 4(b)).

Next, WT, TLR4^−/−^, and TLR9^−/−^ mice were fed with high-casein diet (Ctrl) or high-casein diet plus adenine (CRF) for 8 weeks to induce a CRF-related VC model as we previously reported [18]. All mice subjected to CRF had reduced body weight, but there was no significant difference among the three genotype mice (Figure 4(c)). Importantly, calcium content assay and von Kossa staining demonstrated that TLR9 knockout obviously reduced CRF-related VC, while TLR4 knockout had no effect on CRF-related VC (Figures 4(d) and 4(e)).

### 3.5. TLR9 Activation Promotes BMP-2 Expression through Activation of NF-*κ*B Signaling

**Figure 5:**
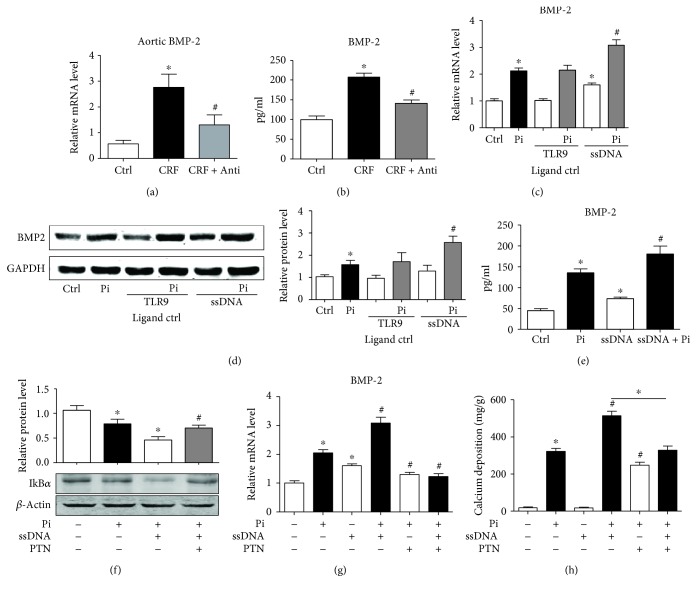
TLR9 activation promotes BMP-2 expression through activation of NF-*κ*B signaling. (a) Real-time PCR analyzed the mRNA levels of BMP-2 in Ctrl, CRF, and CRF + Anti groups. Data are relative to *β*-actin level. *n* = 8~12, ^∗^ *P* < 0.01 vs. Ctrl. ^#^ *P* < 0.01 vs. CRF. (b) Serum BMP-2 levels of Ctrl, CRF, and CRF + Anti groups were analyzed and quantified by ELISA; *n* = 8~12, ^∗^ *P* < 0.01 vs. Ctrl. ^#^ *P* < 0.01 vs. CRF. HASMCs were treated with TLR9 ligand control (1 *μ*g/mL) or ssDNA (10 *μ*g/mL), with or without Pi for 7 days. (c) Real-time PCR analyzed the mRNA level of BMP-2. *n* = 4, ^∗^ *P* < 0.01 vs. Ctrl. ^#^ *P* < 0.01 vs. Pi. (d) Western blot analysis of BMP-2 protein level. GAPDH was a loading control. *n* = 3, ^∗^ *P* < 0.01 vs. Ctrl. ^#^ *P* < 0.01 vs. Pi. (e) HASMCs were treated with Pi or ssDNA for 24 h, and the supernatant BMP-2 was measured by ELISA; *n* = 4, ^∗^ *P* < 0.01 vs. Ctrl. ^#^ *P* < 0.01 vs. Pi. (f-h) HASMCs were treated with Pi, ssDNA, or PTN (PTN was preincubated for 30 minutes, 10 *μ*mol/L) for (f) 30 minutes, and the I*κ*B*α* protein level was measured by western blot. *β*-Actin was a loading control. (g) 24 h, and real-time PCR analyzed the mRNA levels of BMP-2. *n* = 4, ^∗^ *P* < 0.05 vs. Ctrl, ^#^ *P* < 0.05 vs. Pi. (h) 7 days, the calcium content was measured. *n* = 4, ^∗^ *P* < 0.05 vs. Ctrl, ^#^ *P* < 0.05 vs. Pi.

BMP-2 is a member of the TGF-*β* superfamily and involved in physiological ossification [20]. BMP-2 induces osteoblastic differentiation by upregulating Msx2 and Cbf*α*-1 and promotes Pi uptake by VSMCs. Real-time PCR and ELISA analyses showed that both aortic *Bmp2* mRNA and serum BMP-2 protein levels were significantly increased in CRF rats, which were inhibited by antibiotic administration (Figures 5(a) and 5(b)). Pi increased *Bmp2* expression in HASMCs, which was further elevated by treatment with ssDNA (Figure 5(c)). Similarly, the BMP-2 protein level in HASMCs and the supernatant increase after treatment with Pi were further elevated by ssDNA (Figures 5(d) and 5(e)).

NF-*κ*B is the downstream mediator of TLR9 signaling [21]. ssDNA aggravated Pi-induced I*κ*B*α* degradation, while preincubation with an NF-*κ*B inhibitor, parthenolide (PTN), prevented Pi + ssDNA-induced I*κ*B*α* degradation (Figure 5(f)). As a result, PTN preincubation inhibited Pi- and Pi + ssDNA-induced BMP-2 expression (Figure 5(g)). And consequently, Pi- and Pi + ssDNA-induced calcium deposition was reduced by PTN treatment (Figure 5(h)).

### 3.6. BMP-2 Mediated TLR9-Exacerbated HASMC Calcification

**Figure 6:**
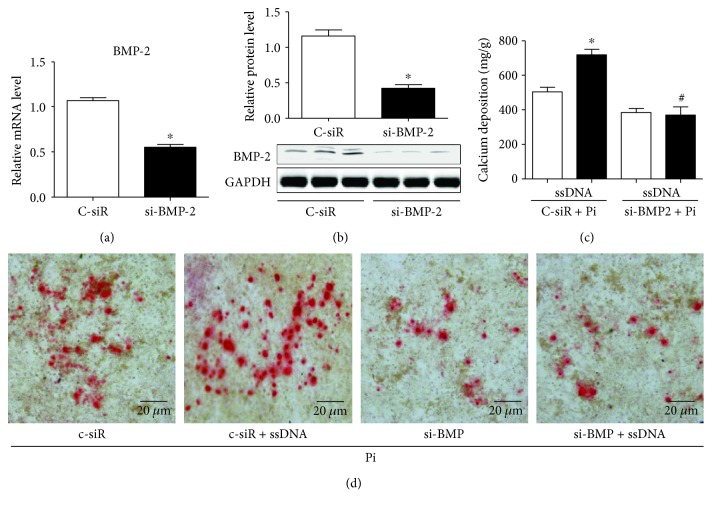
BMP-2 mediated TLR9-exacerbated vascular calcification in HASMCs. (a-b) HASMCs were transfected with scramble siRNA (C-siR) and BMP-2 siRNA (Si-BMP-2) for 2 days. (a) BMP-2 mRNA and (b) protein levels were measured. Data are relative to the *β*-actin level. GAPDH was a loading control. *n* = 3, ^∗^ *P* < 0.01 vs. C-siR. (c-d) HASMCs were transfected with scramble siRNA (C-siR) and BMP-2 siRNA (Si-BMP-2) for 2 days, and then treated with Pi, with or without ssDNA for 7 days. (c) Calcium content was measured. *n* = 3, ^∗^ *P* < 0.05 vs. C-siR + Pi, ^#^ *P* < 0.05 vs. C-SiR + Pi + ssDNA. (d) Alizarin red staining was operated.

To test whether BMP-2 mediated TLR9 activation-promoted VC, *Bmp2* was knocked down by siRNA in HASMCs. Compared with scrambled siRNA (C-siR), *Bmp2* siRNA (si-BMP-2) significantly reduced *Bmp2* mRNA and protein levels (Figures 6(a) and 6(b)). Calcium content assay and Alizarin red staining showed that ssDNA failed to aggravate Pi-induced HASMC calcification after BMP-2 knockdown (Figures 6(c) and 6(d)).

## 4. Discussion

Vascular calcification is the leading cause of cardiovascular events in CRF patients [22]. Systemic inflammation occurs in CRF patients, which is closely related to the morbidity of CRF and cardiovascular events [2]. Here, we identified the role of intestinal bacterial translocation in CRF-related inflammation and VC. Antibiotic supplementation significantly suppressed intestinal bacterial translocation, systemic inflammation, and VC in CRF rats. TLR4 and TLR9 activation in vascular smooth muscle cells (VSMCs) aggravated inorganic phosphate- (Pi-) induced calcification. TLR9 inhibition, but not TLR4 inhibition, reduced CRF-related VC. Furthermore, our data reveals that NF-*κ*B/BMP-2 signaling contributes to TLR9 activation-induced VC.

Recently, the gastrointestinal tract has emerged as a major instigator of systemic inflammation in CRF [3]. More and more studies have explored that intestinal permeability increased in nondialysis CRF rats, and bacteria were observed in the blood and mesenteric lymph node, which was positively correlated with systemic inflammation level in CRF rats, indicating an increased intestinal permeability-induced intestinal bacterial translocation after CRF [4, 23]. Specifically, the evidences of elevated intestinal permeability in CRF patients or animal models include the following: (1) endotoxinemia and bacterial DNA in the intestinal wall, mesenteric lymph nodes, or plasma occur without clinical infection [23, 24]; (2) the intestinal permeability to macromolecules such as polyethylene glycol significantly increased [25, 26]; and (3) gastrointestinal inflammation, including esophagitis, gastritis, duodenitis, and colitis, was found in CRF patients [27]. Thus, increased intestinal permeability is the major cause of systemic inflammation in CRF.

Intestinal bacterial translocation and intestinal flora disorder are distinct concepts. The former is caused by increased intestinal permeability, leading bacteria and its products into the blood, and the latter is the change in the composition or proportion of intestinal flora by pathological factors. In fact, a variety of diseases are associated with intestinal bacterial translocation, including empyrosis, stroke, acute pancreatitis, and cirrhosis of liver. Intestinal bacterial translocation is the main reason for the systemic inflammatory response in these diseases, in which gram-negative bacterial lipopolysaccharide- (LPS-) activated inflammatory response and bacterial DNA caused nonspecific immune response accounting for organ injury such as liver and kidney failure [7–10, 28]. TLR4 and TLR9 are specific receptors for LPS and bacterial DNA, respectively. Thus, we aimed to investigate the effect of inhibiting intestinal bacterial translocation and eliminating bacterial products LPS and DNA by antibiotics on CRF-related VC. Polymycin B sulfate and neomycin sulfate were administered by oral gavage to CRF rats. These two kinds of antibiotics are not absorbed in the intestine, maintaining an intestinal bacterial eliminated state, and significantly reduces the harmful substances produced by intestinal bacteria such as LPS and bacterial DNA [29–33]. 6-week antibiotic administration obviously inhibited intestinal bacterial translocation and CRF-related VC. It also suppressed TLR4 and TLR9 mRNA level in CRF rats' aorta, suggesting that bacterial DNA and LPS might be involved in antibiotic-reduced VC.

The activation of the LPS/TLR4 signal triggers the reactive oxygen species and inflammatory cytokines (IL-8, IL-6, MCP-1, etc.) in arterial endothelial cells and VSMCs, activating vascular inflammation [12]. There is a significant decrease in the risk of atherosclerosis in patients with TLR4 mutation [34]. Su et al. [35] reported that oxidized low-density lipoprotein promoted BMP-2 expression in arterial endothelial cells in a TLR4-dependent manner. However, the role of TLR4 in VSMC calcification, especially in CRF-related VC, is not clear. In this study, our findings reveal that activation of TLR4 by high LPS level in HASMCs promoted Pi-induced VC, while the TLR4 inhibitor failed to block CRF rat serum-augmented VC and TLR4^−/−^ also cannot ameliorate CRF-related VC. Though the LPS level in CRF rats was increased compared with the control group, it was only about 1.5-2 ng/mL. We and others [36] identified that in cultured HASMCs, LPS promoted Pi-induced calcification when its concentration was up to 100 ng/mL, while Isabel et al. used 1000 ng/mL LPS to stimulate human aortic valve interstitial cells to promote BMP-2 expression and bone formation. Thus, in a CRF rat model, it is likely that the LPS concentration was not enough to stimulate VSMCs directly. As LPS-activated M/Ms promoted osteoblastic differentiation and mineralization of CVCs [13], LPS may promote VC through interaction with M/Ms releasing inflammatory cytokines in CRF rats; however, we could not exclude the possibility that low LPS/TLR4 signaling directly promotes VC under chronic stimulation in vivo.

Bacterial DNA/TLR9 signaling is another pathway of bacterial pathogenicity. Recent studies showed that TLR9 activation promotes atherosclerosis [14], vascular endothelial injury [9], macrophage lipid accumulation [37], and myocardial dysfunction [38], suggesting a close relationship between TLR9 signaling and inflammatory vascular diseases. Our findings provide a framework that bacterial DNA significantly increased in CRF rat plasma, activation of TLR9 in HASMCs promoted Pi-induced VC, and TLR9 inhibition with inhibitors or genetic knockout significantly ameliorated CRF-related inflammation and VC, indicating that bacterial DNA/TLR9 signaling plays a key role in CRF-related VC. However, mitochondrial DNA is also a ligand of TLR9; we could not exclude the possibility that mitochondrial DNA plays a role in CRF-related VC. Regardless of the ligands, TLR9 would be a novel target for prevention of CRF-related cardiovascular events.

Usually, TLR9 dimerized when combined with bacterial DNA (containing unmethylated CpG motif), recruiting MyD88. And then interleukin-1 receptor-associated kinase 1 and 4 (IRAK1 and IRAK4) were attracted to MyD88 and phosphorylated and formed a complex with tumor necrosis factor receptor-associated factor 6 (TRAF6), leading to activation of transforming growth factor-activated kinase 1 (TAK1) and finally activation of I*κ*B kinase and NF-*κ*B signaling [39]. NF-*κ*B signaling plays a key role in inducing procalcifying factor BMP-2 expression, which is a member of the TGF-*β* superfamily, involved in physiological ossification and repair of bone [20]. BMP-2 promotes VSMC osteoblastic differentiation by increasing Msx2 and Cbf*α*-1 expression. BMP-2 levels in CRF rat serum and aortic tissue were higher than those in control rats. BMP-2 was induced by TLR9 activation in HASMCs while being blocked by the NF-*κ*B inhibitor. Most importantly, TLR9 activation no longer promoted Pi-induced VC after BMP-2 knockdown, suggesting that TLR9 promoted VC through BMP-2 upregulation.

Uremic toxins play a major role in the pathogenesis of CKD-associated oxidative stress and inflammation [40]. Indoxyl sulfate (IS) and p-cresyl sulfate (PCS), which are protein-bound uremic toxins, increase significantly during kidney injury. The translocation of these toxins from the “leaky gut” into the bloodstream further promotes systemic inflammation, adverse cardiovascular outcomes, and CKD progression [3]. Several studies elucidated that serum uremic toxin levels have a direct relationship with aortic calcification in CKD patients [41, 42]. However, we did not detect serum uremic toxin levels in the present study. We do not exclude the possibility that the antibiotics' effect on CKD-related vascular calcification may partly attribute to decreased uremic toxins levels. And we believe that this is a promising and interesting project that we might research.

The potential therapeutic role of prebiotics and probiotics is being actively studied recently. Vaziri et al. [43] reported that feeding uremic rats with amylose maize-resistant starch (a prebiotic) improved creatinine clearance and reduced kidney inflammation and fibrosis. Small trials in hemodialysis patients have demonstrated that oligofructose-inulin or resistant starch administration obviously reduced circulating indoxyl sulfate and *p*-cresyl sulfate levels and improved gut microbiome [44, 45]. It is possible that probiotics have an inhibitive effect on vascular calcification in CKD patients, but further studies are needed to explore the role.

## 5. Conclusions

Together, our data demonstrates that antibiotics suppress CRF-related VC through clearance of bacterial pathogen and inhibition of TLR9/NF-*κ*B/BMP-2 signaling. The TLR9 signaling pathway might be a novel target for clinical prevention and treatment of vascular calcification and inflammation in CRF patients.

## Data Availability

The data used to support the findings of this study are available from the corresponding author upon request.

## Supplementary Materials

Supplementary Materials Supplement Figure 1: serum renal function factors are ameliorated by antibiotics in adenine-induced CKD rats. (A-D) Serum creatinine, blood urea nitrogen (BUN), calcium (Ca), and Pi levels were measured. (E) Body weight of Ctrl, CRF, and CRF + Anti rats. *n* = 8~12, ^∗^P< 0.05 vs. Ctrl, ^#^P< 0.05 vs. CRF. Supplement Figure 2: bacterial components LPS and DNA contribute to inflammation in macrophages. (A-D) Primary mouse macrophage was preincubated with TLR4 antagonist (LPSRS) and TLR9 inhibitor (ODN TTAGGG) for 30 min and then treated with serum of sham rats or CRF rats for 24 h. Supernatant TNFa, interleukin- (IL-) 6, monocyte chemotactic protein-1 (MCP-1), and IL-12 were determined by BD Cytometric Bead Array. *n* = 4, ^∗^P< 0.05 vs. sham serum + Pi, ^#^P< 0.05 vs. CRF serum + Pi.
