# Supplementary material for: Suppression of Gut Bacterial Translocation Ameliorates Vascular Calcification through Inhibiting Toll-Like Receptor 9-Mediated BMP-2 Expression
Source: Oxid Med Cell Longev. 2019 Mar 17;2019:3415682. doi: 10.1155/2019/3415682 (PMC6441534; doi:10.1155/2019/3415682)

Supplement Figure 1. Serum renal function factors is ameliorated by antibiotics in adenine-induced CKD rats

(A-D) Serum creatinine、blood urea nitrogen(BUN)、calcium(Ca) and Pi levels were measured. (E) Body weight of Ctrl, CRF and CRF+Anti rats. n=8~12, *P<0.05 vs. Ctrl, #P<0.05 vs. CRF.

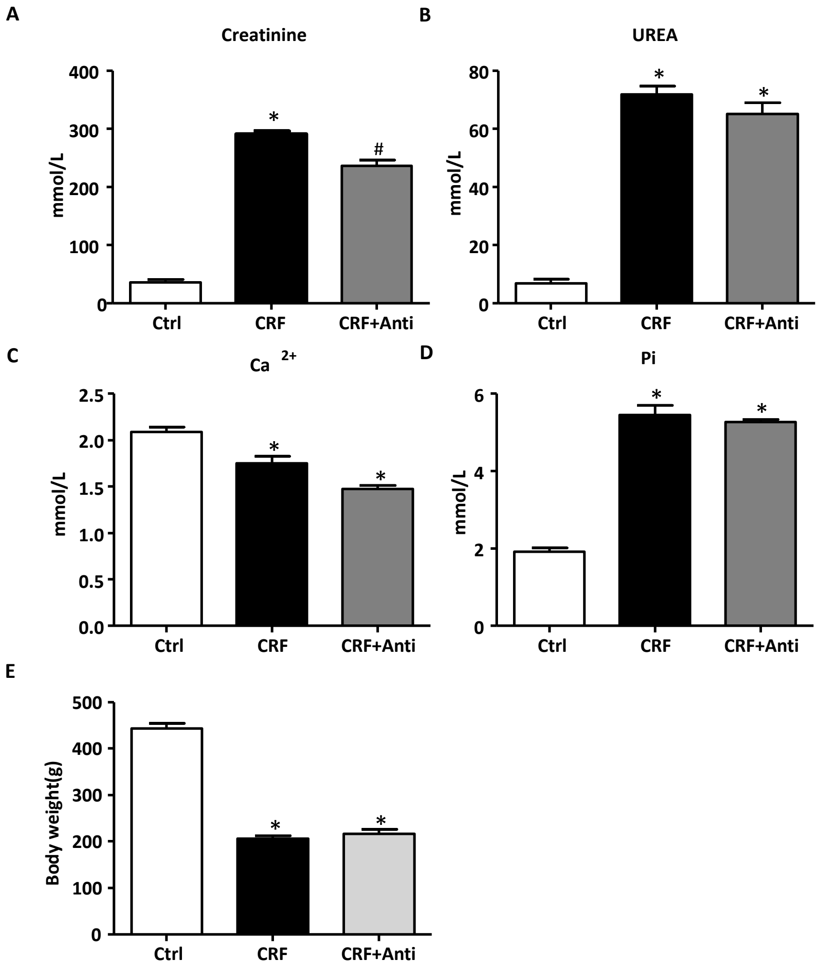

Supplement Figure 2. Bacterial components LPS and DNA contribute to inflammation in macrophages

(A-D) Primary mouse macrophage was pre-incubated with TLR4 antagonist (LPS-RS) and TLR9 inhibitor (ODN TTAGGG) for 30min, and then treated with serum of sham rats or CRF rats for 24h, supernatant TNFα, interleukin (IL)-6, monocyte chemotactic protein-1 (MCP-1), IL-12 were determined by BD Cytometric Bead Array. n=4, *P<0.05 vs. Sham serum+Pi, #P<0.05 vs. CRF serum+Pi.

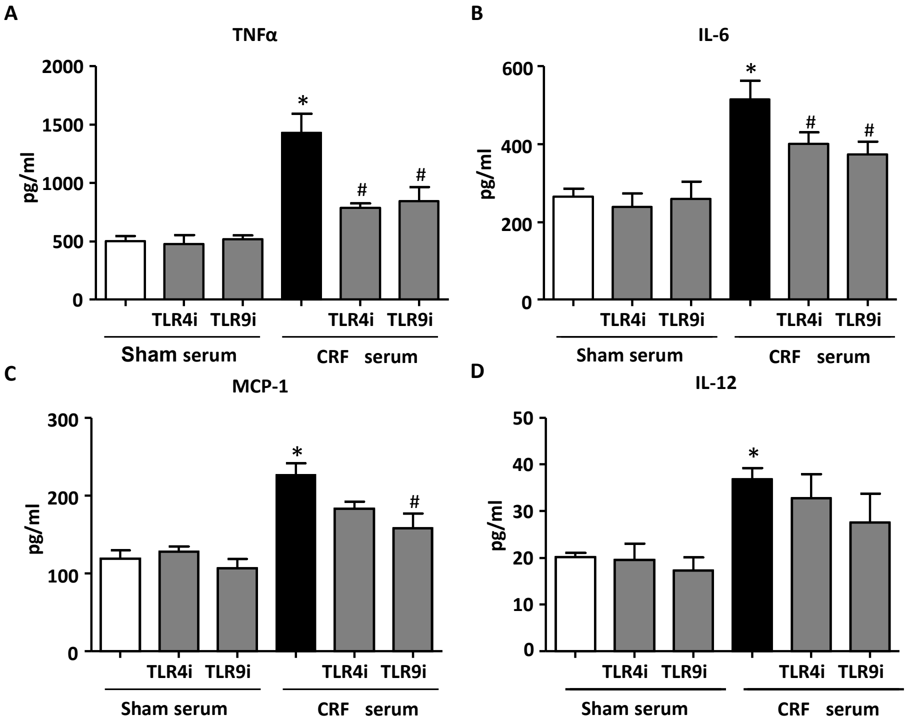

Supplement: Supplementary Materials — Supplement Figure 1: serum renal function factors are ameliorated by antibiotics in adenine-induced CKD rats. (A-D) Serum creatinine, blood urea nitrogen (BUN), calcium (Ca), and Pi levels were measured. (E) Body weight of Ctrl, CRF, and CRF + Anti rats. n = 8~12, ∗P< 0.05 vs. Ctrl, #P< 0.05 vs. CRF. Supplement Figure 2: bacterial components LPS and DNA contribute to inflammation in macrophages. (A-D) Primary mouse macrophage was preincubated with TLR4 antagonist (LPSRS) and TLR9 inhibitor (ODN TTAGGG) for 30 min and then treated with serum of sham rats or CRF rats for 24 h. Supernatant TNFa, interleukin- (IL-) 6, monocyte chemotactic protein-1 (MCP-1), and IL-12 were determined by BD Cytometric Bead Array. n = 4, ∗P< 0.05 vs. sham serum + Pi, #P< 0.05 vs. CRF serum + Pi. [file 3415682.f1.pdf]
